# Improved Gene Fusion Detection in Childhood Cancer Diagnostics Using RNA Sequencing

**DOI:** 10.1200/PO.20.00504

**Published:** 2022-01-27

**Authors:** Jayne Y. Hehir-Kwa, Marco J. Koudijs, Eugene T. P. Verwiel, Lennart A. Kester, Marc van Tuil, Eric Strengman, Arjan Buijs, Mariëtte E. G. Kranendonk, Laura S. Hiemcke-Jiwa, Valerie de Haas, Ellen van de Geer, Wendy de Leng, Jasper van der Lugt, Philip Lijnzaad, Frank C. P. Holstege, Patrick Kemmeren, Bastiaan B. J. Tops

**Affiliations:** ^1^ Princess Máxima Center for Pediatric Oncology, Utrecht, the Netherlands; ^2^ Department of Laboratories, Pharmacy and Biomedical Genetics, Section of Genome Diagnostics, University Medical Center Utrecht, Utrecht, the Netherlands; ^3^ Department of Laboratories, Pharmacy and Biomedical Genetics, Section Pathology, University Medical Center Utrecht, Utrecht, the Netherlands

## Abstract

Gene fusions play a significant role in cancer etiology, making their detection crucial for accurate diagnosis, prognosis, and determining therapeutic targets. Current diagnostic methods largely focus on either targeted or low-resolution genome-wide techniques, which may be unable to capture rare events or both fusion partners. We investigate if RNA sequencing can overcome current limitations with traditional diagnostic techniques to identify gene fusion events.

**METHODS:**

We first performed RNA sequencing on a validation cohort of 24 samples with a known gene fusion event, after which a prospective pan-pediatric cancer cohort (n = 244) was tested by RNA sequencing in parallel to existing diagnostic procedures. This cohort included hematologic malignancies, tumors of the CNS, solid tumors, and suspected neoplastic samples. All samples were processed in the routine diagnostic workflow and analyzed for gene fusions using standard-of-care methods and RNA sequencing.

**RESULTS:**

We identified a clinically relevant gene fusion in 83 of 244 cases in the prospective cohort. Sixty fusions were detected by both routine diagnostic techniques and RNA sequencing, and one fusion was detected only in routine diagnostics, but an additional 24 fusions were detected solely by RNA sequencing. RNA sequencing, therefore, increased the diagnostic yield by 38%-39%. In addition, RNA sequencing identified both gene partners involved in the gene fusion, in contrast to most routine techniques. For two patients, the newly identified fusion by RNA sequencing resulted in treatment with targeted agents.

**CONCLUSION:**

We show that RNA sequencing is sufficiently robust for gene fusion detection in routine diagnostics of childhood cancers and can make a difference in treatment decisions.

## BACKGROUND

Chromosomal rearrangements in the genomes of tumor cells can lead to the formation of chimeric transcripts or gene fusions. It is estimated that gene fusions play a key role in the tumorigenesis and metastasis in 20% of all human cancers.^1^ Some gene fusions occur across multiple cancer types, for example, involving *ALK*, *ROS1*, and *RET*, whereas other gene fusions are more specific and unique to distinct cancer types (eg, *EWSR1-FLI1* fusions in Ewing sarcomas and *KIAA1549*-*BRAF* in pilocytic astrocytomas).^2-4^ Within pediatric oncology, hematologic cancers and sarcomas are characterized by numerous potential fusions.^1,5,6^ The robust detection of gene fusions in diagnostic laboratories is essential to determine an accurate diagnosis and the patient's prognosis and to identify potential therapeutic targets.

**CONTEXT**

**Key Objective**
We investigate if whole transcriptome RNA sequencing can overcome current limitations with traditional techniques to identify gene fusion events within a diagnostic setting in a diverse cohort of patients.
**Knowledge Generated**
Classical gene fusion detection methods are often limited because of resolution or specificity issues. RNA sequencing identified 83 gene fusions in 244 patients, of which 24 were additionally detected in comparison with existing diagnostic procedures (increase of 38%). Several of these additionally detected fusions had added clinical value (prognosis and/or treatment). In addition, data generated by RNA sequencing hold promise for future diagnostic applications, such as expression analysis and tumor classification.
**Relevance**
The use of gene fusion detection with RNA sequencing can significantly increase the diagnostic yield of gene fusion detection.

Gene fusion detection in a diagnostic setting poses several challenges: (1) specific gene fusions may be rare,^7-9^ (2) breakpoints may be atypical,^10-13^ and (3) fusion partners may be promiscuous.^14^ Both reverse transcriptase polymerase chain reaction (RT-PCR)–based assays and fluorescence in situ hybridization (FISH) assays using break-apart probes are a sensitive, but time-consuming method to detect chromosomal rearrangements. Furthermore, because of the location of the probe, atypical fusions might be missed.^15,16^ Both methods are also targeted by design and lack flexibility to robustly detect rare events and/or novel fusion genes. Genome-wide approaches used in routine diagnostics to detect structural variation include karyotyping and single nucleotide polymorphism (SNP) arrays. Karyotyping, however, has a limited resolution not suitable to detect local rearrangements,^17,18^ whereas SNP arrays are unable to detect balanced rearrangements.^19-21^ Therefore, for most cancer samples, multiple methods are performed in parallel to detect clinically relevant gene fusions. Most of these challenges and technical limitations are mitigated by targeted RNA sequencing (RNA-seq) approaches, which allow testing of multiple fusion genes and detection of novel fusion partners. Nevertheless, current implementations of targeted RNA-seq are usually based on gene fusion panels, designed to capture a specific set of gene fusion events for a specific tumor type.^22-24^

Sequencing of total RNA can identify chimeric fusion transcripts genome-wide, providing a potential single assay that maintains the flexibility required to detect rare gene fusion events and atypical breakpoints across a broad variety of tumor types. However, RNA-seq has its own limitations, including technical artifacts that can result in false positives and poor sensitivity for detecting lowly expressed gene fusions or fusions diluted by accompanying noncancerous cells within the sample.^25,26^ The impact of these limitations on RNA-seq–based gene fusion detection in cancer diagnostics is currently unknown. Here, we report the clinical implementation of sequencing ribo-depleted RNA for the identification of chimeric fusion transcripts in a prospective heterogeneous cohort of pediatric cancers.

## METHODS

We created a prospective cohort of consecutive patients entering our center with a suspected neoplasm. Consent was obtained for diagnostic procedures, and RNA could be extracted for 244 patients. The samples included hematologic tumors (n = 97), solid tumors (n = 55), sarcoma (n = 37), brain tumors (n = 41), and suspected neoplasm (n = 14). In parallel to the RNA-seq, existing diagnostic protocols were applied to the samples on the basis of clinical indication (forming the reference standard), including SNP array, karyotyping, FISH, RT-PCR, and in selected cases, targeted RNA-seq (Data Supplement). RNA-seq was performed on fresh (frozen) tissue or bone marrow. In summary, total RNA was isolated and the generated libraries were sequenced on a NovaSeq 6000 (Illumina, San Diego, CA). After aligning the sequence data, gene fusion detection was performed using Star fusion (version 1.6.0)^27^ and annotated using a custom pipeline. The resulting list of potential relevant gene fusions was manually assessed and curated (Data Supplement).

## RESULTS

**FIG 1.**
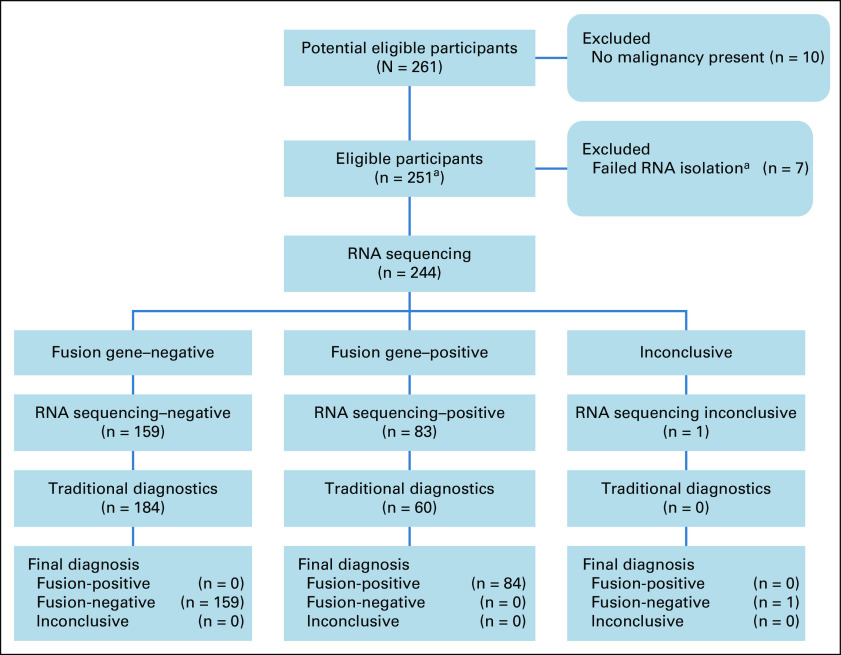
RNA sequencing performance evaluation design and flow of participants. In total, 261 participants were included in the prospective cohort, after excluding samples for which no malignancy was present and RNA failed to be isolated; a total of 244 individuals had samples analyzed in parallel for gene fusions using RNA sequencing and traditional diagnostic methods. ^a^On the basis of sample isolation during the period of the RNA-seq performance evaluation, we estimate that 2.7% of samples were excluded because of insufficient RNA.

**FIG 2.**
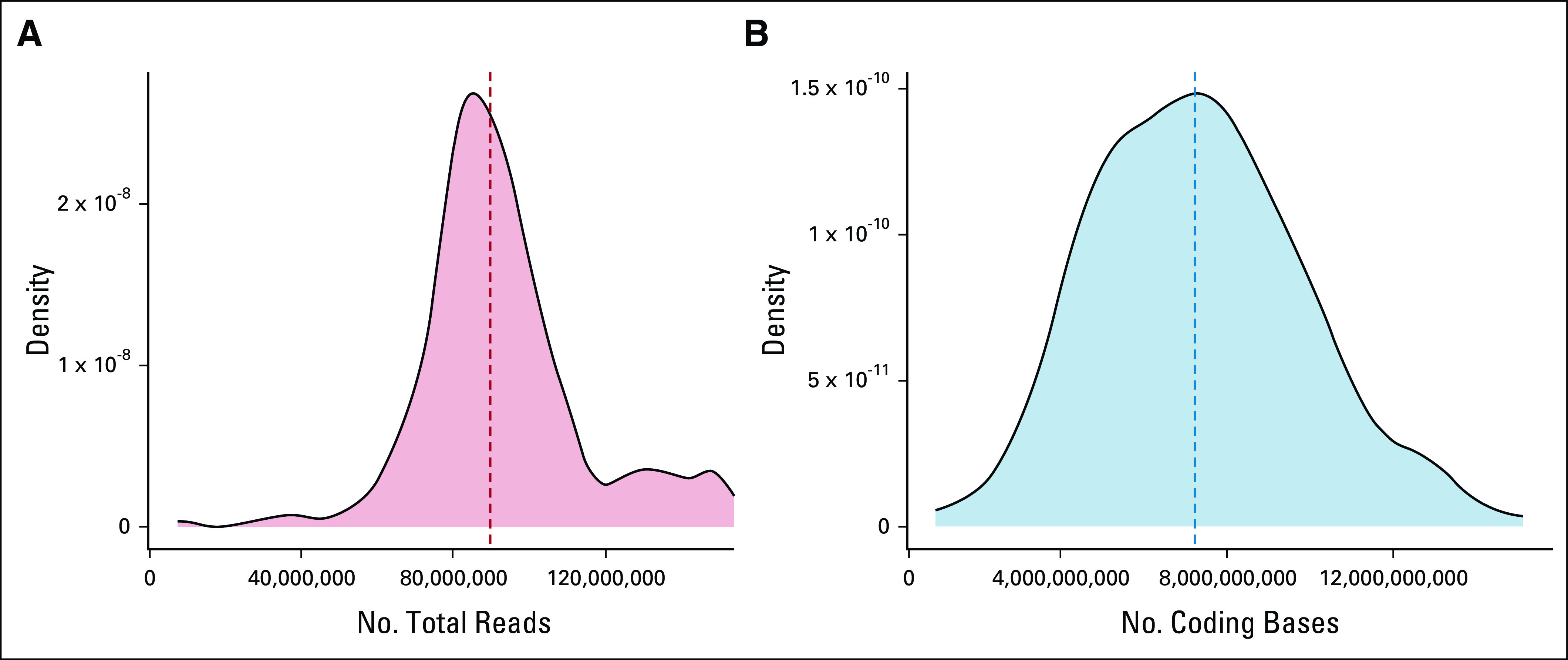
(A) Total number of aligned reads and (B) number of coding bases of all samples in the prospective cohort. A median of 89.6 million of aligned reads were generated covering 7.2 gigabases per sample.

We first screened a small cohort of 24 samples, harboring 25 protein-protein coding fusions to validate and optimize our pipeline (Data Supplement), and then we prospectively sequenced samples in parallel to existing routine diagnostic procedures. In total, we received 261 diagnostic requests. Samples clearly not containing any malignancy were excluded (n = 10), and seven samples were excluded because no RNA could be extracted (n = 7; Fig 1). However, for most of the requests, samples were available to perform RNA-seq (244 of 251 > 97%). We generated a median of 89.6 million uniquely mapped reads and 7.2 gigabases of coding sequence per sample (Fig 2). This cohort reflects the diagnostic case spectrum in our center, including samples taken at diagnosis, from patients confirmed or suspected of having cancer, and relapse samples. The samples were broadly divided into sarcomas, solid tumors, brain tumors, hematologic disorders, and suspected neoplasms, ultimately diagnosed as noncancerous. Similarly, the estimated percentage of neoplastic cells per sample was highly variable (Data Supplement). The performance evaluation of RNA sequencing did not require specific research ethics committee approval as stated in the EU Clinical Trials Directive (2001/20/EC) because it was assessed to be service improvement.

### Diagnostic Yield

In total, we identified 78 clinically relevant protein-protein coding fusions (median fusion fragments per million of 0.57; range of 0.01-34.35); see the Data Supplement for recurrence analysis. Both RNA-seq and routine diagnostics identified 55 of the 78 clinically relevant protein-protein coding fusion events. However, in several cases, traditional methods only identified the clinically relevant 3′ fusion partner and not the 5′ fusion partner. Hence, although the RNA-seq results were concordant with traditional diagnostics, the RNA-seq data provided additional information by identifying the fusion partner. In case PMABM000BJK/27, a *COL6A2-USP6* fusion was identified in routine diagnostics by targeted RNA-seq (Archer FusionPlex Sarcoma, Invitae, Boulder, CO) but not identified using RNA-seq. Further inspection showed that this fusion transcript was present with a fusion fragments per million < 0.1, and neither *COL6A2* nor *USP6* were initially on the inclusion list. The fusion was therefore filtered out of the results. Subsequent rectification and reanalysis results in the correct identification of this fusion. In the remaining 23 discordant cases, the RNA-seq workflow identified protein-protein coding fusion transcripts not identified using traditional methods. These RNA-seq–specific gene fusion events were validated when possible by an independent technique (Data Supplement). This equated to a concordance rate > 98% (55 of 56) with traditional diagnostics and an increase in diagnostic yield from 23% (56 of 244) to 32% (78 of 244; Fig 1), effectively increasing the diagnostic yield of gene fusion detection by 39% (78 of 56).

**FIG 3.**
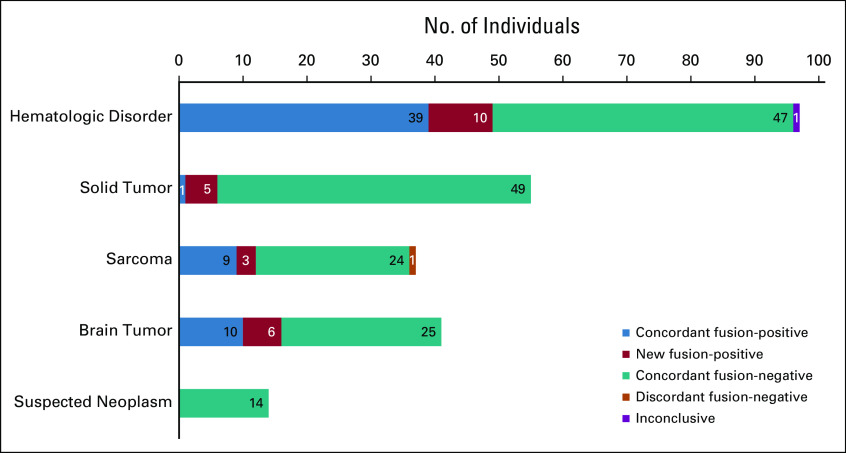
Diagnostic yield of gene fusion detection using RNA-Seq. A prospective cohort of 244 individuals with a pediatric cancer was tested for gene fusion events using RNA sequencing and was divided into the following groups: hematologic disorders (n = 97), solid tumors (n = 55), sarcomas (n = 37), brain tumors (n = 41), and suspected neoplasm (n = 14). The results of the gene fusion detection were then compared with the results of traditional diagnostic techniques. Using RNA sequencing to detect gene fusions and improve pediatric cancer diagnostics.

**FIG 4.**
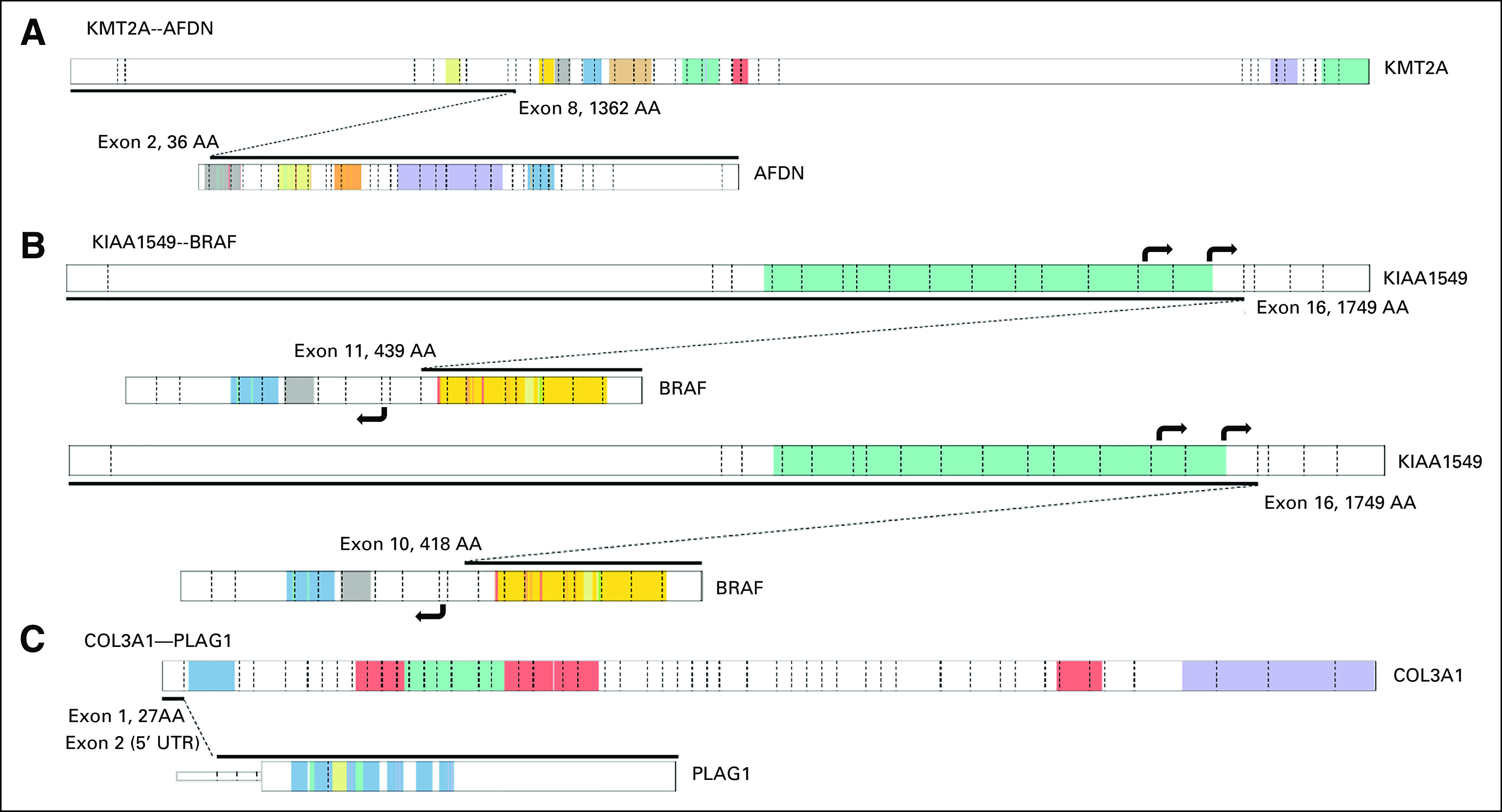
Schematic overview of a selection of fusion genes not detected by routine diagnostic testing. The fusion between the depicted transcripts is indicated by the black line. Exon boundaries are indicated by dashed lines, where known protein domains are colored. Details of protein domains can be found at PeCan Data Portal.^28^. Visualization is based on ProteinPaint (St Jude). (A) *KMT2A-AFDN* fusion transcript not detected by karyotyping and *KMT2A* break-apart FISH, probably because of nonmitosis of leukemic cells or lack of resolution. (B) Two *KIAA1549-BRAF* fusion transcripts not detectable by RT-PCR as performed in routine diagnostics. Primes for RT-PCR are located in KIAA1549 exons 15 and 16 and *BRAF* exon 9 (black arrows). *BRAF* exon 9 is not present in the indicated fusion transcripts and thereby not amplified by RT-PCR. (C) In two lipoblastoma samples, identical *COL3A1-PLAG1* fusion transcripts were detected, where exon 1 of *COL3A1* is fused with the noncoding exon 2 of *PLAG1* (5′UTR). Both samples were negative for this fusion by FISH (break-apart *PLAG1*). FISH, fluorescence in situ hybridization; RT-PCR, reverse transcriptase polymerase chain reaction.

When subdivided by patient groups, 39% of brain tumors, 51% of hematologic samples, 33% of sarcomas, 11% of solid tumors, and 0% of suspected neoplasm samples were fusion-positive on the basis of the RNA-seq data (Fig 3). Of the 23 protein-protein coding fusions additionally identified through RNA-seq, 16 fusions were classified as false negatives; these fusions were not detected using traditional diagnostic techniques because of suboptimal resolution or test design (Data Supplement and Fig 4) or could have been detected using existing assays had these tests been requested during routine diagnostics. Examples include the *EML4-NTRK3* fusion detected by RNA-seq in an infantile fibrosarcoma (IF) sample. Histologically, this case resembled dermatofibrosarcoma protuberans although IF was considered a differential diagnosis. An *ETV6* split FISH was requested,^29^ but yielded negative results because of *EML4* being the 5′ fusion partner.^30^ The *KMT2A-AFDN* fusion detected in an acute myeloid leukemia sample was readily identified by RNA-seq, but undetected in routine diagnostics, probably because of the low resolution of karyotyping or nonmitosis of leukemic cells (Fig 4A). Two pilocytic astrocytoma samples were false negatives for the *KIAA1549-BRAF* fusion because of suboptimal design of the RT-PCR primer positions (Fig 4B), and the *COL3A1-PLAG1* fusion was missed by FISH in two lipoblastoma samples (Fig 4C). The reason for the latter is unclear since a similar fusion was identified in routine diagnostics in another lipoblastoma sample. The remaining seven fusions were not detected because of the lack of a suitable diagnostic assay to detect these events and were labeled undetectable (Data Supplement). These results show that RNA-seq–based gene fusion detection is an unbiased and genome-wide approach that can overcome limitations of targeted and low-resolution assays for all tumor types (Fig 3).

### Promoter and Enhancer Fusions

In addition to the 78 clinically relevant protein-protein fusion events identified by RNA-seq, we found indications for *IGH* promoter fusions in four samples: *IGH-MYC* (n = 2), *IGH-CEBPB*, and *IGH-IGF2BP1*,^31-33^ and a T-cell receptor promoter fusion, *TAL1-TRDC*.^34^ In general, promoter and/or enhancer fusions do not result in chimeric transcripts per se and may not be identified by our RNA-seq workflow. However, in four cases, a chimeric transcript between *IGH* and *CASC11* (upstream of *MYC*), *CEBPB*, and *AC091133.2* (upstream of *IGF2BP1*) were detected. Both *IGH-MYC* fusions were also identified by *MYC* break-apart FISH probes and *IGH-IGF2BP1* by karyotyping. We also detected a fusion with the promoter region of *TRDC* driving expression of *TAL1*, which was also detected by karyotyping. Including these promoter fusions in our results, the increase in diagnostic yield of RNA-seq compared with traditional methods decreases from 39% (78 of 56) to 38% (83 of 60) since most promoter/enhancer fusions (4/5) were also detected by traditional methods.

### Added Value of RNA-seq

We further analyzed the added clinical value of the 24 RNA-seq–specific gene fusions. Seventeen fusions confirmed or corresponded to the initial diagnosis (Data Supplement), for example, the *COL3A1-PLAG1* fusion in a lipoblastoma and the *KIAA1549-BRAF* fusions in two pilocytic astrocytomas. Seven events modified the diagnosis and/or possible treatment of the patient. For example, case PMABM000CCU/104 is an acute megakaryoblastic leukemia (FAB M7) in which a *CBFA2T3-GLIS2* was detected. This could have affected patient care had it been detected at disease presentation^35,36^ since stem-cell transplantation is considered early on as a treatment option for leukemias harboring this fusion. Several other RNA-specific fusions are tyrosine kinase fusions. These have previously been described in (pediatric) tumors, although their incidence is very low, and therefore not always routinely tested. However, these tyrosine kinase fusions are known (putative) targets for targeted agents and could potentially widen treatment options. Case PMABM000CZK/193 resembled dermatofibrosarcoma protuberans histologically, but the *EML4-NTRK3* fusion established this case as an IF. This patient was subsequently enrolled in a clinical trial with larotrectinib. Similarly, case PMABM000BQE/7, in which a *ZCCHC8-ROS1* fusion was identified, received targeted treatment.

### *ZCCHC8-ROS1*–Positive Glioma

**FIG 5.**
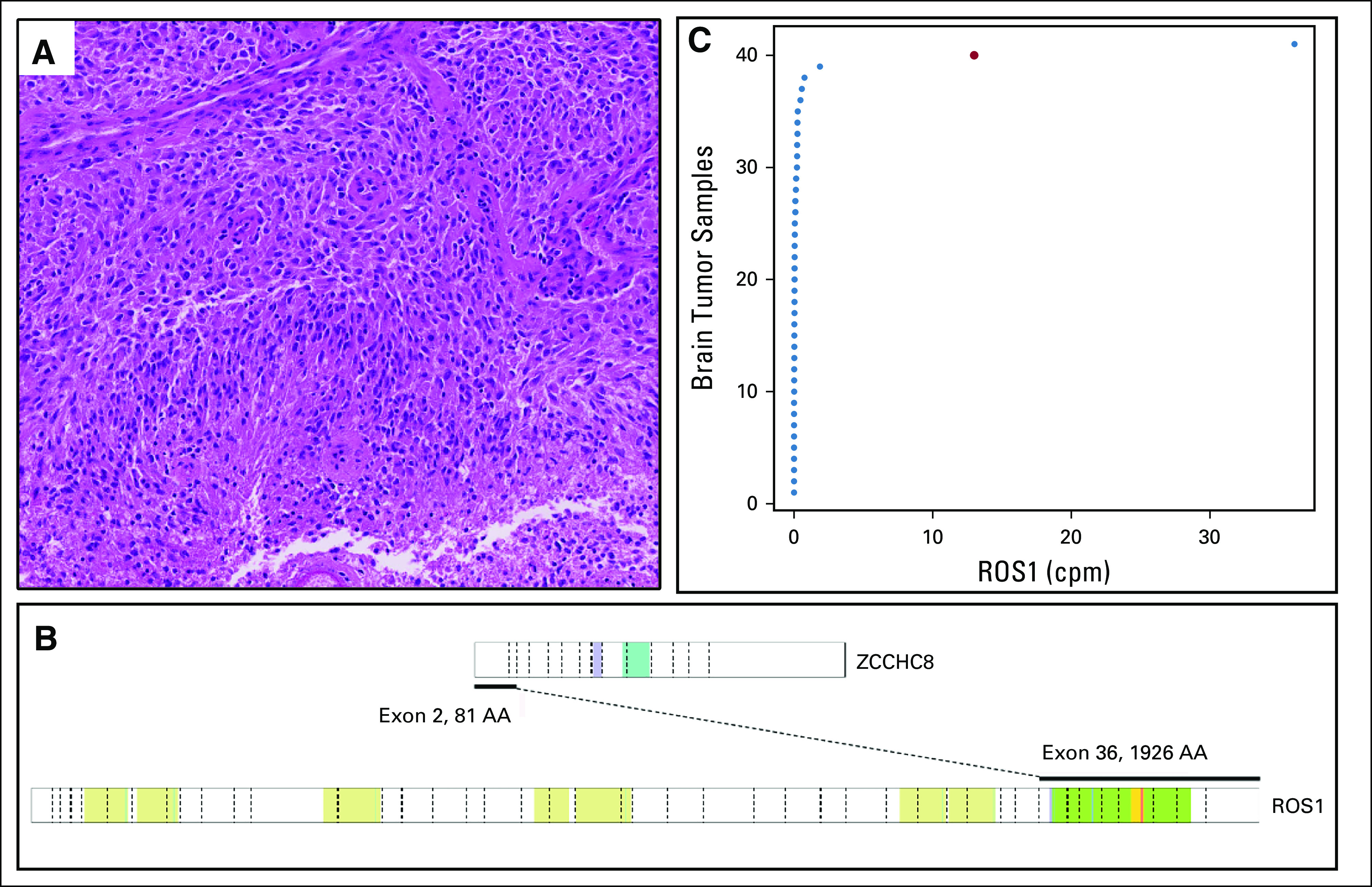
(A) Hematoxylin and eosin staining (magnification 50×). (B) Schematic representation of the *ZCCHC8-ROS1* fusion transcript, where exon 2 of *ZCCHC8* is fused to exon 36 of the *ROS1* gene. Colored regions indicate different protein domains (for legend, see PeCan Data Portal^28^). (C) RNA expression of *ROS1* in all brain tumors analyzed within this cohort. The high-grade glioma harboring the *ZCCHC8-ROS1* rearrangement shows the second highest *ROS1* expression (red dot), likely as a consequence of the rearrangement. On the *x*-axis, the cpm sequence reads mapped to the *ROS1* gene are plotted per brain tumor sample. cpm, counts per million.

Case PMABM000BQE/7, a one-year-old boy, presented at diagnosis with vomiting and a drooping right eyelid. The magnetic resonance imaging of the brain revealed a large mixed solid-cystic, intra-axial mass, in the right frontal region of the brain, with profound hydrocephalus and debulking surgery being performed. Histology of the tissue (Fig 5A) showed a glial tumor with marked atypia, increased mitotic activity, florid microvascular proliferation, and palisading tumor necrosis, consistent with glioblastoma with a detrimental prognosis. However, methylation profiling classified the tumor as an infantile hemispheric glioma.^37^ This tumor type is characterized by targetable kinase fusions and a better outcome than glioblastoma.^38^ RNA-seq identified a *ZCCHC8-ROS1* fusion (Fig 5B). A break-apart *ROS1* FISH resulted in a difficult to interpret patterns and failed to confirm the fusion. We hypothesized that *ZCCHC8*, as a 5′ fusion partner, might drive (over)expression of the *ROS1* transcript and compared the *ROS1* expression with that of all other brain tumor samples in our cohort. The tumor's *ROS1* expression is the second highest (Fig 5C). All brain tumors, with the exception of a teratoma sample, show lower *ROS1* expression, supporting the validity of the *ZCCHC8-ROS1* fusion.

Shortly after surgery, chemotherapy was commenced according to the HGG HIT infant protocol (cyclophosphamide, vincristine, high-dose methotrexate, carboplatin, and etoposide), but switched to BBSFOP HGG^39^ after 6 weeks because of local progression. Fifteen months after the start of the BBSFOB protocol, local and metastatic progression was observed.^39^ The Lansky score of the patient was 100, and after discussion with the parents, consent was given to use entrectinib. Entrectinib (F06 formulation) was started at the recommended phase II dose of 300 mg/m^2^ once a day, and the medication was tolerated well. A magnetic resonance imaging was performed 1 month after commencing therapy and showed a decrease in the solid component. The patient remained stable on entrectinib for almost a year but showed progression thereafter.

## DISCUSSION

We present the results of detecting clinically relevant fusion transcripts using RNA-seq on a prospective pediatric pan-cancer cohort (Table 1). The RNA-seq performance evaluation was completed in parallel to existing diagnostic methodologies. We show that RNA-seq has the same specificity as current diagnostic methods and a higher sensitivity, increasing the diagnostic yield by 38%. Using a single unbiased genome-wide assay, we detected a broad range of gene fusion events involving 73 different gene partners.

**TABLE 1.**
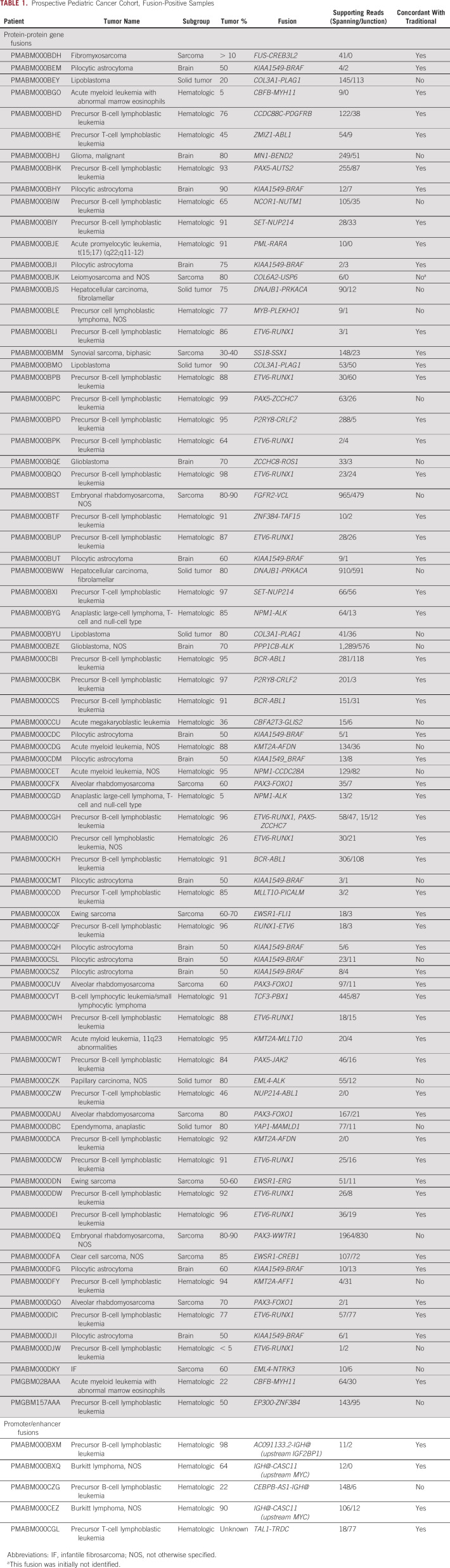
Prospective Pediatric Cancer Cohort, Fusion-Positive Samples

Most RNA-seq–specific fusions that were specifically identified by RNA-seq independently confirm a histologic diagnosis, but a subset of fusions identified were highly relevant and could affect treatment outcome. Examples are the *CBFA2T3-GLIS2* fusion (acute megakaryoblastic leukemia, FAB M7)^35,36^ and the five druggable events (depending on clinical trials or compassionate use): *ZCCHC8-ROS1* (high-grade glioma), *PPP1CB-ALK* (high-grade glioma), *EML4-ALK* (papillary thyroid carcinoma), *FGFR2-VCL* (embryonal rhabdomyosarcoma), and *EML4-NTRK3* (IF). Novel treatments targeting these events could have a significant impact on clinical management, highlighted by the patient with the *ZCCHC8-ROS1*–positive glioma that was treated with entrectinib. This demonstrates the power of RNA-seq in routine diagnostics.

In addition to the 78 protein-protein coding gene fusion events, we also detected *IGH* and *TCR* rearrangements in five samples despite the data analysis not being optimized to detect promoter and enhancer fusions. Additional validations are needed to show if all promoter fusions can reliably be detected by RNA-seq, but this is probably an advantage over targeted assays that are unlikely to detect these rearrangements because of the size of the breakpoint regions. Although not all promoter/enhancer fusions may result in chimeric transcripts, the effect of these fusions, for example, (over)expression of the 3′ fusion partner, is likely to manifest itself in the RNA-seq data, highlighting additional tests that could be developed: One could think of quality controls in the analysis pipeline to screen for potential fusion events that result in overexpression (eg, the *ZCCHC8-ROS1* fusion, Fig 5C) or develop algorithms that classify tumor entities on the basis of their expression profiles. This could reveal novel tumor entities,^40^ not readily diagnosed using classical methods, or improve diagnostics similar to the methylation-based classifier used in neuro-oncology.^37^

Although the use of expression values holds promises for the future, there are several more immediate advantages of RNA-seq over traditional diagnostic methods. First, it is more efficient to use a single assay for gene fusion detection than a range of different techniques that require specific expertise and equipment. Furthermore, RNA-seq can detect fusion events not yet known to be clinically relevant. Hence, the assay itself does not have to be redesigned when novel fusion biomarkers are introduced into the clinic, in contrast to targeted approaches that will need to be tested, validated, and implemented.^41,42^ Second, both fusion partners are identified, in contrast to when break-apart FISH assays are used. This is relevant for tumor entities where specific fusion partners determine the diagnosis, like soft tissue sarcomas; for example, *EWSR1-FLI1* is indicative for Ewing sarcoma, *EWSR1-ATF1* for clear cell sarcoma, and *EWSR1-DDIT3* for myxoid liposarcoma. In the future, even fusion partners of druggable targets might become relevant predicters of therapy response.^43-45^ Third, RNA-seq identifies both fusion partners and the exact breakpoint sequence (Data Supplement). This information is indispensable when developing patient-specific assays to determine disease dissemination and minimal residual disease, for example, in patients with lymphoma and leukemia.^24,46-49^ This is especially valuable when rare fusions are identified for which no predesigned (commercial) diagnostic test is available.

Although RNA-seq provides clear advantages over current diagnostic methods, it also has limitations: (1) We deliberately chose to use fresh (frozen) material for RNA isolation. Although this is normal in hemato-oncology (fresh bone marrow or peripheral blood), in tissue diagnostics, it is standard practice to use formalin-fixed, paraffin-embedded material to isolate DNA and/or RNA. Instead of adapting our RNA-seq workflow to compensate for the suboptimal quality and yield of formalin-fixed, paraffin-embedded tissue–derived RNA, we opted to change the tissue processing workflow in our center. This resulted in a high percentage of fresh frozen tissue being available for RNA-seq (> 97%), and when fresh frozen material is available, this resulted in RNA of sufficient quantity and quality to perform RNA-seq in 243 of 244 samples. Since our center is the only centralized center for pediatric oncology in the Netherlands, this was relatively easy to implement, but we realize that this is more difficult for referral laboratories. (2) Our assay requires an input of 300 ng of total RNA, although as little as 50 ng can be used for small biopsies. This is still more than the amount required for some assays, for example, 10 ng for an RT-PCR or a 4 μm tissue slice for FISH analysis. However, since we had insufficient or low-quality RNA to perform RNA-seq in only 3% of cases (n = 7 + 1), in our daily practice, this is not a major issue. For cases with low amounts of RNA, it is still possible to use targeted fusion gene detection methods. (3) Some fusions are more readily detected than others, depending on the expression level of the specific fusion and the neoplastic cell content of the sample. For example, *KIAA1549-BRAF* fusions are lowly expressed. As a result, these fusions would normally be filtered out had it not been for an inclusion list in our analysis pipeline. Similarly, fusions lowly expressed or in samples with a low percentage of neoplastic cells are likely to be missed if data analysis is not specifically adapted to detect these fusions. For example, the *COL6A2-USP6* fusion that was initially missed by RNA-seq because of low fusion fragments per million (< 0.1) combined with the absence of *COL6A2* or *USP6* on the inclusion list. (4) Finally, RNA-seq has a longer turnaround time of 7-21 days compared with more targeted approaches.

On the basis of the results described in this paper, we perform RNA-seq for all patient samples in our center. For now, we will continue the workflow as described here, RNA-seq in parallel to classical methods. One reason for this is that some clinical study protocols requiring specific (classical) diagnostic tests, but we also aim to define the minimum number of reads required for a reliable RNA-seq result. We are confident that RNA-seq will replace many of the classical tests routinely performed in our center in the future.

We show the advantages of detecting fusion transcripts using RNA-seq compared with current diagnostic approaches. It detects both fusion partners and breakpoint regions for follow-up assays and yields data that could advance future diagnostic testing. More importantly, it has a significantly higher diagnostic yield than current diagnostic assays and can be easily implemented in routine diagnostics.

## Data Availability

Sequences of all gene fusion breakpoints detected via RNA-seq are provided in the Data Supplement. The RNA-seq data, subject to consent, are available in the EGA data set EGAD00001007701.
